# New Cytotoxic Cembranolides from the Soft Coral *Lobophytum michaelae*

**DOI:** 10.3390/md10020306

**Published:** 2012-01-31

**Authors:** Shang-Kwei Wang, Chang-Yih Duh

**Affiliations:** 1 Department of Microbiology, Kaohsiung Medical University, Kaohsiung 807, Taiwan; Email: skwang@cc.kmu.edu.tw; 2 Department of Marine Biotechnology and Resources, National Sun Yat-Sen University, Kaohsiung 804, Taiwan; 3 Asia-Pacific Ocean Research Center, National Sun Yat-Sen University, Kaohsiung 804, Taiwan

**Keywords:** *Lobophytum michaelae*, cembranolides, cytotoxicity

## Abstract

Six new cembranolides, michaolides L–Q (**1**–**6**), and a known cembranolide, lobomichaolide (**7**) were isolated from the CH_2_Cl_2_ extract of the soft coral *Lobophytum michaelae*. Their structures were established by extensive spectral analysis. The anti-HCMV (human cytomegalovirus) activity of **1**–**7** and their cytotoxicity against selected cell lines were evaluated.

## 1. Introduction

**Figure 1:**
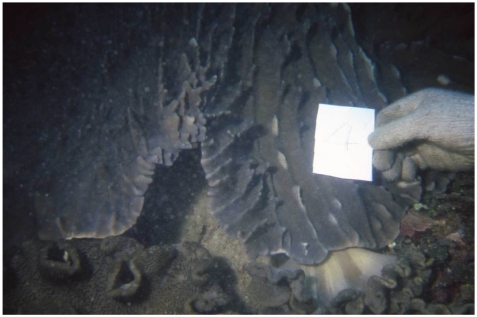
Soft coral *Lobophytum michaelae*.

**Figure 2:**
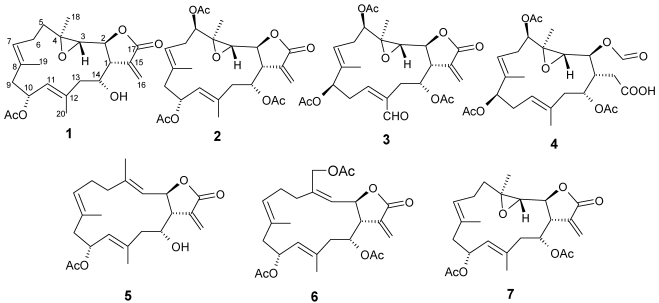
Structures of compounds **1**–**7**.

Soft corals of the genus *Lobophytum* (Alcyoniidae) have been reported as a rich source of secondary metabolites endowed with a range of structural diversity and various biological activities [1,2,3,4,5,6,7,8,9,10,11,12,13,14,15,16,17,18,19,20,21,22,23,24]. Previous bioassay results of some cembranoids and their analogues have demonstrated remarkable pharmacological potential such as cytotoxicity against various cancer cell lines [2,3,4,5,6,7,8,9], anti-inflammatory properties [11,12,13], antimicrobial activities [11], and HIV-inhibitory activity [14]. In previous papers [2,3,4], we reported the isolation of several cytotoxic cembranolides, lobomichaolide, crassolide, and michaolides A–K from samples of the soft coral *Lobophytum michaelae* Tixier-Durivault (Alcyoniidae) (Figure 1). In this report, a new specimen of the soft coral *L. michaelae* was studied since the CH_2_Cl_2_ solubles exhibited significant cytotoxity against HT-29 (human colon adenocarcinoma) and P-388 (mouse lymphocytic leukemia) cell lines as determined by standard procedures. [25,26] Bioassay-guided fractionation of the extract resulted in the isolation of six new cembranolides, michaolides L–Q (**1**–**6**), together with the known cembranolide, lobomichaolide (**7**) [2,3] (Figure 2).

## 2. Results and Discussion

Michaolide L (**1**) was isolated as a colorless oil, [α]_D_^25^ +13.3 (*c* 0.1, CHCl_3_). HRESIMS, ^13^C NMR, and DEPT spectroscopic data established the molecular formula of **1** as C_22_H_30_O_6_. The IR spectrum of **1** indicated the presence of the functionalities of ester group(s) (*ν*_max_ 1734 cm^−1^) and an α-methylene-γ-lactone (*ν*_max_ 1766, 1668 cm^−1^). The presence of the α-methylene-γ-lactone system in **1** was also demonstrated by UV absorption at 222 (log ε 3.68) nm and signals at δ 5.67 (H-16) and 6.44 (H-16) in the ^1^H NMR spectrum (Table 1). The ^1^H NMR spectrum of **1** also showed signals for two olefinic protons at δ 5.65 (H-11), and 5.19 (H-7) ppm; four oxymethine protons at δ 4.46 (t, *J* = 6.8 Hz, H-2), 2.70 (d, *J* = 6.8 Hz, H-3), 5.64 (m, H-10), and 4.38 (m, H-14); one methine proton at δ 2.90 (m, H-1), two olefinic methyl groups at δ 1.55 (H_3_-19) and 1.72 (H_3_-20); and a methyl group in acetate ester at δ 2.05. HMBC spectrum exhibited a methyl-bearing trisubstituted epoxide [δ_H_ 2.70 (d, *J* = 6.8 Hz, H-3), 1.42(H_3_-18); δ_C_ 59.6 (CH), 64.0 (qC), 20.4 (CH_3_)] (Table 1). The spectral data of **1** indicated some similarities to those of lobomichaolide (**7**) [2,3], except for the data due to C-14. The H^1^–H^1^ COSY spectrum exhibited correlations from H-13 to H-3, H-5 to H-7, and H-9 to H-11. ^1^H–^1^H long-range correlations were also observed between H-1 to H_2_-16, H-7 to H_3_-19, and H-11 to H_3_-20. These spectroscopic findings and the nine degrees of unsaturations indicated that **1** was a 14-membered cembrane-type diterpene skeleton with an α-methylene-γ-lactone. 

**Figure 3:**
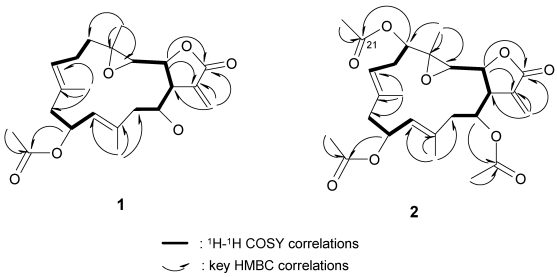
COSY and HMBC correlations of compounds **1** and **2**.

**Figure 4:**
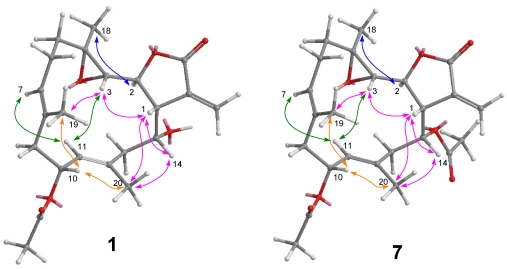
NOESY correlations of compounds **1** and **7**.

**Figure 5:**
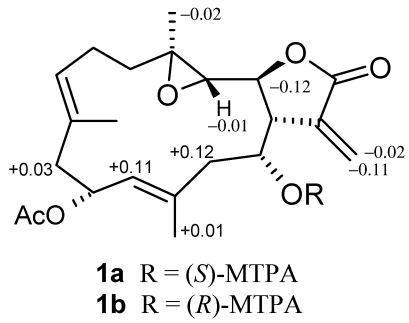
Absolute stereochemistry of **1**: Δδ*_S–R_* values in ppm for MTPA esters **1a** and **1b**.

After assignments between all the C–H bondings were made based on an HSQC experiment, the planar structure was determined by HMBC analysis. The correlations according to HMBC are shown in Figure 3. The stereochemistry for the trisubstituted olefins of **1** was determined by NOESY analysis. The NOESY correlations between H-7 and H-9, and H-11 and H-13 disclosed the *E* configurations for the trisubstituted olefins. The chemical shift values at δ_C_ 15.6 and 15.9 (for C-19 and C-20, respectively) also supported the *E* configurations [2,3]. The NOESY correlations (Figure 4) observed between H-3 and H-1/ H-11/H_3_-19, H-14 and H-1/H_3_-20, H-7 and H-9/ H-11, H-10 and H_3_-20/H_3_-19, and H_3_-18 and H-2 indicated the relative configurations for the 14-membered ring carbons, which were identical to those of lobomichaolide (**7**). Analysis of the Δδ*_S–R_* values (Figure 5) according to the Mosher model pointed to an *R* configuration for C-14 of **1**, because H_2_-13, H-11, and Me-20 of (*S*)-MTPA ester **1a** were less shielded by the phenyl ring of MTPA products. Therefore, the absolute stereochemistry of Michaolide L (**1**) was established as (1*R*,2*S*,3*S*,4*R*,10*S*,14*R*,7*E*,11*E*)-10-acetoxy-14-hydroxy-3,4-epoxycembra-7,11-dien-17,2-olide ambiguously.

**Table 1:** ^1^H and ^13^C NMR data for compounds **1**–**3**.

Position	1		2		3	
	δ_H_ (*J* in Hz) *^a^*	δ_C_^*b*^	δ_H_ (*J* in Hz) *^a^*	δ_C_^*b*^	δ_H_ (*J* in Hz) *^a^*	δ_C_^*b*^
1	2.90 m	48.1	3.07 m	44.1	2.97 m	45.2
2	4.46 t (6.8) *^c^*	76.7	4.68 t (6.8)	75.2	4.52 t (6.8)	75.3
3	2.70 d (6.8)	59.6	2.83 d (6.8)	60.3	2.76 d (6.8)	59.9
4		64.0		62.7		63.4
5	1.91 m	23.8	4.83 dd (10.8, 3.2)	75.5	5.05 dd (11.2, 3.6)	74.3
6	2.03 m, 2.18 m	33.5	2.21 m, 2.42 m	30.3	2.33 m, 2.54 m	29.1
7	5.19 br d (8.0)	129.5	5.10 t (5.6)	122.5	5.46 t (6.4)	121.7
8		127.5		132.3		131.3
9	2.18 m, 2.45 m	44.7	2.38 m	44.5	5.25 br s	75.3
10	5.64 m	67.8	5.67 m	68.4	2.82 m, 3.27 m	29.4
11	5.65 m	127.8	5.45 m	128.1	6.73 dd (9.6, 4.4)	147.0
12		137.5		135.8		137.8
13	2.48 m, 2.91 m	44.2	2.37 m, 2.51 m	41.8	2.28 m, 2.89 m	34.7
14	4.38 m	68.6	5.40 m	71.1	5.77 m	69.3
15		136.7		134.9		136.1
16	5.67 m, 6.44 d (2.8)	122.3	5.72 s, 6.40 s	124.0	5.75 d (2.8), 6.39 d (2.8)	124.7
17		169.4		168.9		168.4
18	1.42 s	20.4	1.25 s	14.7	1.49 s	15.7
19	1.55 s	15.6	1.66 s	16.5	1.67 s	12.9
20	1.72 s	15.9	1.83 s	16.0	10.14 s	190.3
5-OAc			2.01 s	170.1	2.13 s	170.1
				21.1		21.2
9-OAc					2.13 s	169.8
						20.8
10-OAc	2.05 s	170.5	2.05 s	170.2		
		21.4		21.2		
14-OAc			2.11 s	170.3	2.02 s	169.8
				21.3		20.5

*^a^* 400 MHz in CDCl_3_ (assigned by COSY, HSQC, and HMBC experiments); *^b^* 100 MHz in CDCl_3_ (assigned by DEPT, COSY, HSQC, and HMBC experiments); *^c^* *J* values (Hz) in parentheses.

**Figure 6:**
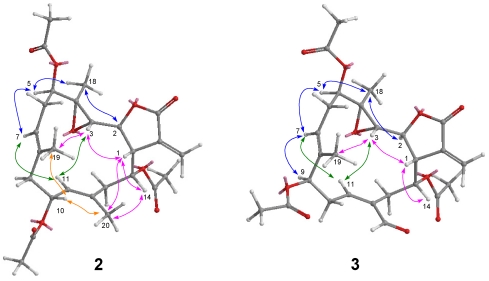
NOESY correlations of compounds **2** and **3**.

Michaolide M (**2**) was shown to have the molecular formula of C_24_H_34_O_9_ by HRESIMS and from its ^13^C NMR data. The ^1^H and ^13^C NMR spectral data (Table 1) of **2** closely resembled those of **7** except for the signals at C-5. ^1^H–^1^H COSY cross peak (Figure 3) between H-5 and H-6/H-7 as well as HMBC correlations (Figure 3) between H-5 and C-6/C-4/C-21 revealed the presence of an additional acetoxyl [δ_H_ 4.83 (dd, *J* = 10.8, 3.2, H-5), δ_C_ 75.5 (CH, C-5), 170.1 (qC), 21.2 (CH_3_)] at C-5 in **2**. NOESY correlations (Figure 6) between H-5 and H-7, H-3 and H-1/ H-11/H_3_-19, H-14 and H-1/H_3_-20, H-7 and H-9/H-11, H-10 and H_3_-20/H_3_-19, and H_3_-18 and H-2 indicated the relative configurations for **2** resembled those of **7** except for the additional C-5 (*R*) acetoxy.

**Figure 7:**
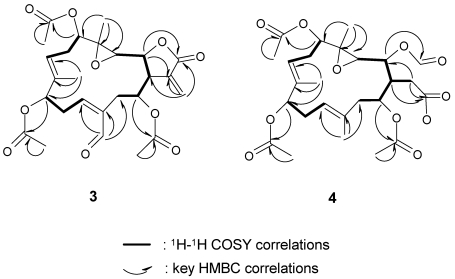
COSY and HMBC correlations of compounds **3** and **4**.

Michaolide N (**3**) analyzed for C_26_H_32_O_10_ from its HRESIMS and NMR spectroscopic data. The NMR features of compound **3** were analogous to those of **2** with exception that the secondary acetoxyl attached to C-10 was shifted to C-9 and the methyl attached to C-12 was replaced by an aldehyde [δ_H_ 10.14, δ_C_ 190.3] (Table 1). ^1^H–^1^H COSY cross peaks (Figure 6) between H-9 and H-10/H-11 as well as HMBC correlations (Figure 7) between H-20 and C-11/C-12/C-13 as well as between H_3_-19 and C-7/C-8/C-9 helped to ascertain these assignments. The relative stereochemistry of **3** was determined by NOESY correlations (Figure 6) between H-7 and H-5/H-9/H-11, H-3 and H-1/H-11, H-14 and H-1/H-20, H-10 and H-20, H_3_-18 and H-2, and H-11 and H_2_-13.

**Figure 8:**
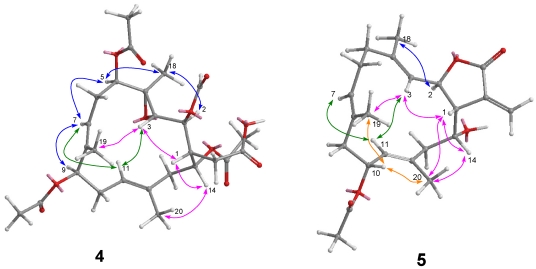
NOESY correlations of compounds **4** and **5**.

Michaolide O (**4**) had the molecular formula, C_26_H_36_O_11_. Detailed comparison of the ^1^H and ^13^C NMR spectral data (Table 2) of **4** and **3** revealed that **4** differed from **3** at C-20 and the α-exo-methylene-γ-lactone moiety. A COSY correlation (Figure 7) from H-1 to H_2_-15 and HMBC correlations (Figure 7) from H_2_-15 to C-16/C-2 and from H-2 toC-17 revealed that the α-exo-methylene-γ-lactone moiety in **3** was oxidized to a formyloxyl (δ_H_ 8.27 s, δ_C_ 161.6) at C-2 and carboxylmethyl at C-1 in **4**. The relative stereochemistry of **4** was determined by NOESY correlations (Figure 8) between H-7 and H-5/H-9/H-11, H-3 and H-1/H-11/H_3_-19, H-14 and H-1/H_3_-20, H_3_-18 and H-2, and H-11 and H_2_-13. 

**Table 2:** ^1^H and ^13^C NMR data for compounds **4**–**6**.

Position	4		5		6	
	δ_H_ (*J* in Hz) *^a^*	δ_C_*^b^*	δ_H_ (*J* in Hz) *^a^*	δ_C_*^b^*	δ_H_ (*J* in Hz) *^a^*	δ_C_*^b^*
1	2.61 m	43.2	2.68 br s	48.0	2.79 m	46.1
2	4.52 t (6.8)*^c^*	76.4	5.38 dd (8.8, 2.7)	73.5	5.53 dd (8.7, 6.8)	72.5
3	2.82 d (6.8)	60.2	5.03 d (8.8)	123.3	5.18 m	127.2
4		63.4		140.0		140.6
5	4.89 dd (10.8, 3.6)	75.1	2.30 m	24.1	2.33 m	24.1
6	2.82 m, 2.55 m	29.8	2.21 m	37.9	2.36m, 2.40 m	33.1
7	5.34 m	120.1	4.96 m	129.7	4.93 m	128.8
8		133.5		130.6		131.1
9	5.16 m	76.6	2.32 m, 2.39 m	44.5	2.29 m, 2.40 m	44.5
10	2.37 m, 2.48 m	41.8	5.65 m	68.5	5.66 m	68.0
11	5.30 m	123.4	5.16 d (9.1)	126.6	5.19 m	127.7
12		131.7		134.5		136.8
13	2.31 m, 2.68 m	43.8	2.21 m, 2.43 m	45.5	2.28 m, 2.53 m	41.8
14	5.32 m	69.2	4.15 m	72.9	5.28 m	74.0
15	3.75m, 3.85 m	35.2		136.8		136.6
16		175.6	5.65 s, 6.41 s	122.7	5.70 s, 6.37 s	124.4
17	8.26 s	161.6		167.8		167.3
18	1.48 s	15.7	1.80 s	17.3	4.55 d (12.6), 4.91 d (12.6)	62.3
19	1.60 s	12.3	1.64 s	16.7	1.61 s	16.6
20	1.74 s	15.6	1.75 s	16.3	1.81 s	16.0
5-OAc	2.11 s	170.1				
		21.1				
9-OAc	2.11 s	170.3				
		21.2				
10-OAc			2.04 s	170.0	2.01 s	170.2
				21.4		21.0
14-OAc	2.12 s	170.3			2.02 s	170.8
		21.3				21.3
18-OAc					2.03 s	169.3
						20.9
16-O*H*	6.18 brs					

*^a^* 400 MHz in CDCl_3_ (assigned by COSY, HSQC, and HMBC experiments); *^b^* 100 MHz in CDCl_3_ (assigned by DEPT, COSY, HSQC, and HMBC experiments); *^c^* *J* values (Hz) in parentheses.

**Figure 9:**
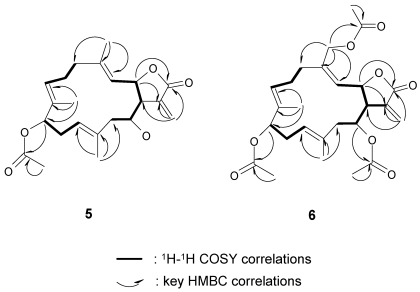
COSY and HMBC correlations of compounds **5** and **6**.

Michaolide P (**5**) was shown to have the molecular formula of C_22_H_30_O_5_ by HRESIMS and from its ^13^C NMR data. The ^1^H and ^13^C NMR spectral data (Table 2) of **5** closely resembled those of **1** except for the replacement of the trisubstituted epoxy by a trisubstituted olefin at Δ^3^. HMBC correlations (Figure 9) between H_3_-18 and C-3/C-4/C-5 confirmed the presence of a trisubstituted olefin at C-3. The relative stereochemistry was determined by NOESY correlations (Figure 8) between H-3 and H-1/H-11/H_3_-19, H-14 and H-1/H_3_-20, H-7 and H-9/ H-11, H-10 and H_3_-20/H_3_-19, and H_3_-18 and H-2.

Michaolide Q (**6**) had the molecular formula of C_26_H_34_O_8_ by HRESIMS and from its ^13^C NMR data. The ^1^H and ^13^C NMR spectroscopic data (Table 2) of **6** closely resembled those of **5** except for the signals at C-18 and C-14. The low field chemical shift and HMBC correlations (Figure 9) between H_2_-18 and C-3/C-4/C-5/C-21 confirmed the presence of an acetoxy group at C-18. HMBC correlations (Figure 8) between H-14 and C-22 revealed the presence of a second acetoxy group at C-14. The relative stereochemistry was determined by NOESY correlations between H-3 and H-1/H-11/H_3_-19, H-14 and H-1/H_3_-20, H-7 and H-9/H-11, H-10 and H_3_-20/H_3_-19, and H_3_-18 and H-2.

The cytotoxicity toward P-388 (mouse lymphocytic leukemia), HT-29 (human colon adenocarcinoma), A-549 (human lung epithelial carcinoma) tumor cells, and human embryonic lung (HEL) cells of michaolides L–Q (**1**–**6**) and lobomichaolide (**7**) were shown in Table 3. Non-cytotoxic cembranoid, michaolide O (**4**) was tested for anti-HCMV activity and showed a negative result (IC_50_ > 200 μM/mL). The α-exo-methylene-γ-lactone moiety is important for cytotoxicity by comparing the cytotoxicity of **4** with those of **1**–**3**, **5**, and **6** [4]. The absolute stereochemistry of the known cembranolide, lobomichaolide (**7**) [2,3] should be drawn as in Figure 2 since cembranolides **1** and **7** both exhibited positive optical rotations.

**Table 3:** Cytotoxicityof **1**–**7**.

Compounds	Cell Lines ED_50_ (μM/mL)			
	A549	HT-29	P-388	HEL
**1**	1.2	0.8	0.3	1.0
**2**	2.0	4.9	1.5	3.2
**3**	2.1	1.6	0.4	2.0
**4**	61.3	61.5	39.6	60.2
**5**	3.2	2.8	2.0	2.9
**6**	2.0	1.5	1.0	1.8
**7**	1.9	1.4	0.4	1.7

## 3. Experimental Section

### 3.1. General Experimental Procedures

Optical rotations were determined with a JASCO P1020 digital polarimeter. Ultraviolet (UV) and infrared (IR) spectra were obtained on JASCO V-650 and JASCO FT/IR-4100 spectrophotometers, respectively. NMR spectra were recorded on a Varian MR 400 NMR spectrometer at 400 MHz for ^1^H and 100 MHz for ^13^C. ^1^H NMR chemical shifts are expressed in δ (ppm) referred to the solvent peaks δ_H_ 7.27 for CDCl_3_, and coupling constants are expressed in Hz. ^13^C NMR chemical shifts are expressed in δ (ppm) referred to the solvent peaks δ_C_ 77.0 for CDCl_3_. ESI-MS were recorded by ESI FT-MS on a Bruker APEX II mass spectrometer. Silica gel 60 (Merck, Germany, 230–400 mesh) and LiChroprep RP-18 (Merck, 40–63 μm) were used for column chromatography. Precoated silica gel plates (Merck, Kieselgel 60 F_254_, 0.25 mm) and precoated RP-18 F_254s_ plates (Merck) were used for thin-layer chromatography (TLC) analysis. High-performance liquid chromatography (HPLC) was carried out using a Hitachi L-7100 pump equipped with a Hitachi L-7400 UV detector at 220 nm together with a semi-preparative reversed-phase column (Merck, Hibar LiChrospher RP-18e, 5 μm, 250 × 25 mm).

### 3.2. Biological Material

The soft coral *L. michaelae* Tixier-Durivault (Alcyoniidae) was collected at Ken-Ting, Ping-Tong County, Taiwan, in June 2002, at a depth of 3–4 m and was stored for 2 weeks in a freezer until extraction. Identification was kindly verified by Prof. Keryea Soong, Institute of Marine Biology, National Sun Yat-sen, Taiwan. A voucher specimen, MR-004, was deposited in the Department of Marine Biotechnology and Resources, National Sun Yat-sen University, Taiwan.

### 3.3. Extraction and Isolation

The bodies of the soft coral *L. michaelae* were freeze dried to give 1.10 kg of a solid, which was extracted with CH_2_Cl_2_ (3.0 L × 3). After removal of solvent in vacuo, the residue (20 g) was chromatographed over Si gel 60 using *n*-hexane and *n*-hexane/EtOAc mixtures of increasing polarity. Elution with *n*-hexane/EtOAc (49:1) gave fractions containing **7**, with *n*-hexane/EtOAc (9:2) gave fractions containing **2** and **6**, with *n*-hexane/EtOAc (5:2) gave fractions containing **3**, with *n*-hexane/EtOAc (3:2) gave fractions containing **4**, with *n*-hexane/EtOAc (1:1) gave fractions containing **1** and **5**. Compounds **1**–**7** were further purified by RP-18 HPLC eluting with MeOH/H_2_O (50:50), MeOH/H_2_O (76:24), MeOH/H_2_O (70:30), MeOH/H_2_O (66:34), MeOH/H_2_O (50:50), MeOH/H_2_O (76:24), and MeOH/H_2_O (78:22), respectively.

Michaolide L (**1**): White amorphous powder (5 mg); [α]_D_^25^ +13.3 (*c* 0.1, CHCl_3_); UV λ_max_ (MeOH) nm (log ε): 222 (3.68); IR (neat) *ν*_max_ 3412, 1766, 1734, 1668 cm^−1^; ^1^H NMR (CDCl_3_, 400 MHz) and ^13^C NMR (CDCl_3_, 100 MHz) data in Table 1; HRESIMS *m/z* 413.1916 [M + Na]^+^ (calcd. for C_22_H_30_O_6_Na, 413.1914).

Preparation of Mosher’s Esters of **1**. In separate NMR tubes, duplicate (1.0 mg) samples of **5** were dissolved in 0.6 mL of pyridine-*d*_5_ and allowed to react for 3 h at room temperature with (*R*)- and (*S*)-MTPA chloride (one drop) to yield (*S*)-MTPA ester **1a** and (*R*)-MTPA ester **1b**, respectively. Selected ^1^H NMR (pyridine-*d*_5_, 300 MHz) of **1a**: ^1^H NMR (CDCl_3_, 300 MHz): δ 1.35 (3H, s, H_3_-18), 1.51 (3H, s, H_3_-19), 1.98 (3H, s, H_3_-20), 2.08 (3H, s, 10-OAc), 2.32 (1H, dd, *J* = 12.1, 8.9 Hz, H-9), 2.56 (1H, d, *J* = 12.1 Hz, H-9), 2.86 (2H, m, H-13), 3.02 (1H, d, *J* = 6.1 Hz, H-3), 3.54 (3H, s, OMe), 4.57 (1H, t, *J* = 6.9 Hz, H-2), 5.22 (1H, br d, *J* = 8.2 Hz, H-7), 5.93 (1H, d, *J* = 8.4 Hz, H-11), 6.05 (1H, d, *J* = 2.8 Hz, H-16), 6.12 (1H, ddd, *J* = 10.8, 6.0, 1.5 Hz, H-14), 6.47 (1H, d, *J* = 3.2 Hz, H-16), 7.41–7.61 (5H, m, Ph). Selected ^1^H-NMR (pyridine-*d*_5_, 300 MHz) of **1b**: ^1^H NMR (CDCl_3_, 300 MHz): δ 1.37 (3H, s, H_3_-18), 1.52 (3H, s, H_3_-19), 1.97 (3H, s, H_3_-20), 2.08 (3H, s, 10-OAc), 2.32 (1H, dd, *J* = 12.3, 9.4 Hz, H-9), 2.53 (1H, d, *J* = 12.3 Hz, H-9), 2.74 (2H, m, H-13), 3.03 (1H, d, *J* = 6.8 Hz, H-3), 3.53 (3H, s, OMe), 4.69 (1H, t, *J* = 6.6 Hz, H-2), 5.20 (1H, br d, *J* = 6.9 Hz, H-7), 5.82 (1H, d, *J* = 8.8 Hz, H-11), 6.14 (1H, d, *J* = 2.2 Hz, H-16), 6.58 (1H, dd, *J* = 11.4, 3.8 Hz, H-14), 6.58 (1H, d, *J* = 3.2 Hz, H-16), 7.42–7.62 (5H, m, Ph).

Michaolide M (**2**): White amorphous powder (3 mg); [α]_D_^25^ +11.2 (*c* 0.1, CHCl_3_); UV λ_max_ (MeOH) nm (log ε): 221 (3.67); IR (neat) *ν*_max_ 1765, 1736, 1728, 1669 cm^−1^; ^1^H NMR (CDCl_3_, 400 MHz) and ^13^C NMR (CDCl_3_, 100 MHz) data in Table 1; HRESIMS *m/z* 489.2102 [M + Na]^+^ (calcd. for C_24_H_34_O_9_Na, 489.2101).

Michaolide N (**3**): White amorphous powder (1 mg); [α]_D_^25^ +7.6 (*c* 0.1, CHCl_3_); UV λ_max_ (MeOH) nm (log ε): 220 (3.76); IR (neat) *ν*_max_ 2820, 2730, 1765, 1736, 1726, 1669 cm^−1^; ^1^H NMR (CDCl_3_, 400 MHz) and ^13^C NMR (CDCl_3_, 100 MHz) data in Table 1; HRESIMS *m/z* 527.1885 [M + Na]^+^ (calcd. for C_26_H_32_O_10_Na, 527.1884).

Michaolide O (**4**): White amorphous powder (2 mg); [α]_D_^25^ +3.1 (*c* 0.1, CHCl_3_); IR (neat) *ν*_max_ 3420, 1740, 1731,, 1712, 1675 cm^−1^; ^1^H NMR (CDCl_3_, 400 MHz) and ^13^C NMR (CDCl_3_, 100 MHz) data in Table 2; HRESIMS *m/z* 547.2155 [M + Na]^+^ (calcd. for C_26_H_36_O_11_Na, 547.2156).

Michaolide P (**5**): White amorphous powder (1 mg); [α]_D_^25^ +122.0 (*c* 0.1, CHCl_3_); UV λ_max_ (MeOH) nm (log ε): 221 (3.96); IR (neat) *ν*_max_ 3450, 1765, 1735,1666 cm^−1^; ^1^H NMR (CDCl_3_, 400 MHz) and ^13^C NMR (CDCl_3_, 100 MHz) data in Table 2; HRESIMS *m/z* 397.1993 [M + Na]^+^ (calcd. for C_22_H_30_O_5_Na, 397.1992).

Michaolide Q (**6**): White amorphous powder (1 mg); [α]_D_^25^ +81.6 (*c* 0.1, CHCl_3_); IR (neat) *ν*_max_ 1762, 1731, 1675 cm^−1^; ^1^H NMR (CDCl_3_, 400 MHz) and ^13^C NMR (CDCl_3_, 100 MHz) data in Table 2; HRESIMS *m/z* 497.2154 [M + Na]^+^ (calcd. for C_26_H_34_O_8_Na, 497.2152).

Lobomichaolide (**7**): Colorless prism (25 mg); m.p. 180–181; [α]_D_^25^ +55.6 (*c* 0.1, CHCl_3_).

### 3.4. Cytotoxicity Assay

Cytotoxicity was determined on P-388 (mouse lymphocytic leukemia), HT-29 (human colon adenocarcinoma), and A-549 (human lung epithelial carcinoma) tumor cells using a modification of the MTT colorimetric method according to a previously described procedure [25,26]. The provision of the P-388 cell line was supported by J.M. Pezzuto, formerly of the Department of Medicinal Chemistry and Pharmacognosy, University of Illinois at Chicago. HT-29 and A-549 cell lines were purchased from the American Type Culture Collection.

### 3.5. Anti-HCMV Assay

To determine the effects of natural products upon HCMV cytopathic effect (CPE), confluent human embryonic lung (HEL) cells grown in 24-well plates were incubated for 1 h in the presence or absence of various concentrations of tested natural products. Then, cells were infected with HCMV at an input of 1000 pfu (plaque forming units) per well of 24-well dish. Antiviral activity was expressed as IC_50_ (50% inhibitory concentration), or compound concentration required to reduce virus induced CPE by 50% after 7 days as compared with the untreated control. To monitor the cell growth upon treating with natural products, an MTT-colorimetric assay was employed [27].

## 4. Conclusion

The α-exo-methylene-γ-lactone moiety is important for cytotoxicity by comparing the cytotoxicity of **4** with those of **1**–**3**, **5**, and **6** [4]. The absolute stereochemistry of the known cembranolide, lobomichaolide (**7**) [2,3] should be drawn as in Figure 2 since cembranolides **1** and **7** both exhibited positive optical rotations.
